# Large changes in detected selection signatures after a selection limit in mice bred for voluntary wheel-running behavior

**DOI:** 10.1371/journal.pone.0306397

**Published:** 2024-08-01

**Authors:** David A. Hillis, Liran Yadgary, George M. Weinstock, Fernando Pardo-Manuel de Villena, Daniel Pomp, Theodore Garland

**Affiliations:** 1 Genetics, Genomics, and Bioinformatics Graduate Program, University of California, Riverside, California, United States of America; 2 Department of Genetics, University of North Carolina at Chapel Hill, Chapel Hill, North Carolina, United States of America; 3 The Jackson Laboratory for Genomic Medicine, Farmington, CT, United States of America; 4 Department of Genetics and Genome Science, University of Connecticut Health Center, Farmington, Connecticut, United States of America; 5 Department of Evolution, Ecology, and Organismal Biology, University of California, Riverside, California, United States of America; Government College University Faisalabad, PAKISTAN

## Abstract

In various organisms, sequencing of selectively bred lines at apparent selection limits has demonstrated that genetic variation can remain at many loci, implying that evolution at the genetic level may continue even if the population mean phenotype remains constant. We compared selection signatures at generations 22 and 61 of the “High Runner” mouse experiment, which includes 4 replicate lines bred for voluntary wheel-running behavior (HR) and 4 non-selected control (C) lines. Previously, we reported multiple regions of differentiation between the HR and C lines, based on whole-genome sequence data for 10 mice from each line at generation 61, which was >31 generations after selection limits had been reached in all HR lines. Here, we analyzed pooled sequencing data from ~20 mice for each of the 8 lines at generation 22, around when HR lines were reaching limits. Differentiation analyses of allele frequencies at ~4.4 million SNP loci used the regularized T-test and detected 258 differentiated regions with FDR = 0.01. Comparable analyses involving pooling generation 61 individual mouse genotypes into allele frequencies by line produced only 11 such regions, with almost no overlap among the largest and most statistically significant peaks between the two generations. These results implicate a sort of “genetic churn” that continues at loci relevant for running. Simulations indicate that loss of statistical power due to random genetic drift and sampling error are insufficient to explain the differences in selection signatures. The 13 differentiated regions at generation 22 with strict culling measures include 79 genes related to a wide variety of functions. Gene ontology identified pathways related to olfaction and vomeronasal pathways as being overrepresented, consistent with generation 61 analyses, despite those specific regions differing between generations. Genes *Dspp* and *Rbm24* are also identified as potentially explaining known bone and skeletal muscle differences, respectively, between the linetypes.

## Introduction

Although evolution can result in organisms with spectacular capabilities or able to survive in exceptionally inhospitable environments, all adaptations are bound within certain limits. These limits are commonly observed in laboratory and agricultural selection experiments [1–5]. Among various possible causes of selection limits [2, 6, 7], the simplest explanation is the loss of genetic variation, such that narrow-sense heritability declines to zero (e.g., see [8]). However, selection experiments have frequently found that genetic variation remains after reaching a selection limit [1, 3, 5, 9–13]. Even for alleles favored by selection, fixation is far from guaranteed [4, 12–14].

One selection experiment that has continued selection long after reaching a limit is the High Runner (HR) mouse experiment, which started in 1993 with the purchase of 224 outbred ICR mice from Harlan Sprague Dawley [15]. These were randomly bred for two generations, then split into ten breeding pairs to found each of eight closed lines. Four of these lines were designated to serve as non-selected control lines, while the other four were selected based on voluntary wheel running. In selected lines, all mice are given access to wheels for 6 days and the male and female of each family with the highest running on days 5 and 6 would be used as breeders (no sib-mating). After about 22 generations of selection, three of the four HR lines (with the fourth line following suit a few generations later) had plateaued in their running at approximately 2.5 to 3 times as many revolutions as the controls [3]. Recently, the experiment has reached its 100^th^ generation since selection began and, with exception of some generations when the experiment moved from Wisconsin to California (generations 32 to 35) and during Covid-19 lockdowns (generations 91 to 98), selection has continued nearly uninterrupted in the interim. Whether selection interruption following the move to California resulted in statistically significant changes to running behavior has not yet been analyzed.

Numerous physiological and morphological differences between the HR and control lines have been documented [16–20]. These include traits associated with motivation to run, such as changes in dopamine [21, 22], serotonin [23], and endocannabinoid signaling [24], as well as changes in brain size and structure [25]. Additionally, changes associated with ability to run have been found, including endurance capacity [26], maximal aerobic capacity (VO_2Max_) [27–31], heart size [28, 32, 33], skeletal muscle physiology [34–37], and bone morphology [38–46].

Previously, whole-genome differentiation analyses using individual mouse data from 10 males from each of the eight lines at generation 61 identified at least 13 genomic regions differentiated between the control and HR lines [13, 47]. Within these regions were genes associated with development of the brain, heart, bones, and limbs, in addition to reward pathways, and even the vomeronasal system (see also [48]). Dropping individual lines from analyses revealed new potential signatures of selection and demonstrated that the HR lines have evolved in different ways at the genomic level (“multiple solutions” [49]) that increase wheel-running behavior [47]. Despite being ~30–35 generations past the selection limit, a great deal of genetic diversity remained in all 8 lines including many regions identified as differentiated between the HR lines and controls.

With the selection limit achieved near generation 22, one might expect many if not most biologically relevant SNPs to already be differentiated by that generation. Thus, with respect to the ability to detect selection signatures, little advantage would be gained from allowing ~30–35 generations to pass before testing for allelic differentiation between the HR and control lines. Furthermore, simulations performed by Baldwin-Brown et al. [50] demonstrate that increasing the number of generations could reduce power to detect some loci under selection, which they attributed to noise created by random genetic drift. Reasonably, one might expect that drift over enough generations may cause control lines to diverge from each other in allele frequencies, such that selection signatures are obscured in statistical tests that compare replicate sets of selected and control lines. For example, if some control lines become fixed for one allele and the remaining control lines become fixed for another, then, even if all HR lines were fixed for the same allele favored by selection, statistically significant differentiation would be difficult to detect. Therefore, analyses of a generation close to when a selection limit is first reached would be optimal for tests of genetic differentiation.

In the present study, we analyze pooled sequence data from each of the four HR lines and four control lines at generation 22. Although these analyses identify many regions containing genes associated with systems known to be phenotypically differentiated between the HR and control linetypes, they largely differ from those previously identified with the generation 61 individual mouse sequence data [13]. Furthermore, the number of differentiated regions detected at generation 22 are more than 20-fold greater than those detected with generation 61 data (treated as pooled data).

We first discuss possible methodological causes of these differences (e.g., pooled vs individual mouse data) and find them lacking. We therefore develop a simple simulation model, with leptokurtic distribution of locus effect sizes, to test the possibility that a hypothetical physiological constraint on wheel running could contribute to the differences between generations 22 and 61 selection signatures. Ignoring locus effect size, results demonstrate that such constraints can contribute to a reduction in power and increased variability in the detected response to selection in generations after the selection limit. However, the magnitude of these effects appears insufficient to explain the differences observed between generations 22 and 61 in the real data. In addition, effect size was an important determinant of the ability to detect selection signatures in the simulations, including a more than 2-fold increase in power to detect loci with large effect size at generation 22 as compared to generation 61. Thus, with strict culling procedures, we suspect that many of the selection signatures detected at both generations are likely to represent loci with relatively large effects on wheel running. The regions detected at generation 22 include genes related to olfactory/vomeronasal systems, which are also identified at generation 61 [13, 47, 48].

## Materials and methods

### High runner mouse model

As described previously [3, 15], 112 males and 112 females of the outbred Hsd:ICR strain were purchased from Harlan Sprague Dawley in 1993 and designated as generation -2. Mice would be randomly bred for 2 generations (-2 and -1) with 2–3 generation -1 mice from each family randomly chosen to contribute to 1 of 8 different closed lines. Four of these lines were randomly picked to be “High Runner” (HR) lines, in which mice would be selected for breeding based on voluntary wheel running. The remaining 4 lines were used as Control (C) lines, without any selection. Generation 0 was the first generation where HR lines were paired based on running levels (10 males and 10 females for each line) with generation 1 the first product of selection.

Wheel running measurements were collected by giving mice at approximately 6–8 weeks of age, access to wheels for six days. The amount of running (total revolutions) on days 5 and 6 was used as the selection criterion. Both days 5 and 6 are used for repeatability in running behavior and robustness against bad data for a single day [3]. For the HR lines, the highest-running male and female from within each of 10 families were chosen as breeders (within-family selection). For the non-selected C lines, one male and one female from each of 10 families were chosen as breeders, independent of wheel running measurements. Sib-mating was disallowed in all lines [15].

### Genome sequencing and allele frequency determination

Roughly 10 male and 10 female mice were taken from each line at generation 22 [20]. Mice were decapitated without anesthesia because blood was being taken for a study of hormone levels (corticosterone) that can respond rapidly to additional handling or anesthesia. Subsequently, their DNA was extracted from tail tips and then pooled for determination of allele frequency for each line. This pooled DNA was sequenced with paired end pooled sequencing with Illumina HiSeq 2500 sequences were trimmed and aligned to the GRCm38/mm10 mouse genome assembly. Generation 22 used trimmomatic v0.39 for trimming, BWA v0.7.17 for alignment, Samtools v1.14 for sorting and indexing, picard v2.26.11 for marking duplicates, and GATK v4.1.8.1 for calling SNPs. SNPs were filtered to keep those with read quality (“RQ”) ≥ 20, DP ≥ 10, were missing either quality score, or missing the allele frequency all together, or had MAF > 0.0126. Allele frequencies (“AF”) were determined for generation 22 by taking the read depth of the alternate nucleotide allele (i.e., allele differing from the GRCm38/mm10 alignment) and dividing by the read depth for the locus. After all quality control methods were implemented, 4,446,523 loci remained for generation 22.

The generation 61 data were taken from Hillis et al. [13]. 80 male mice (10 from each line) were subject to whole genome sequencing and reads were trimmed and aligned to the GRCm38/mm10 mouse genome assembly as described in Didion et al. [51]. This generated an average read depth of 12X per mouse. SNPs were filtered to keep those with genotype quality ("GQ") > 5, read depth (“DP”) > 3, minimum allele frequency (“MAF”) > 0.0126 for all samples, and Mapping Quality ("MQ") > 30. One of the 80 mice was excluded due to likely contamination [as in 52], leaving 79 for the following analyses. SNPs not found to be present in at least two of the 79 mice were also removed from analysis. After all quality control methods were implemented, 5,932,148 loci remained for analyses. To allow comparison with the pooled sequencing data from generation 22, we calculated allele frequencies as the number of alternative alleles divided by 2 times the number of mice (i.e., 20 or 18 for HR3).

### Statistical analyses

For generations 22 and 61 we used an arcsine-squared transformation [53] of the AF. Analyses were conducted on both generations using a traditional T-test, regularized T-test (RegT) [50, see also 54], and a variant of the regularized T-test which uses a sliding window to calculate $\bar{v}$ (WRT test) (S1 File). The regularized T-test was based on a Bayesian method meant to minimize the type-I errors caused by sampling error with small sample sizes [50, 54], such as the 8 total lines in the HR mouse selection experiment. We performed these tests and determined the permutation-based false discovery rate (FDR) for each method (see below). For comparison, we also performed the RegT and WRT tests on loci found in both generation 22 and 61 (from pooling individual mouse genotypes) data sets along with the FDR. Since standard T-tests do not require whole genome or region variances of other loci, the p-values of loci shared between the two generations could simply be extracted from the complete original analyses.

### Permutation-based false discovery rate

To determine relative power of generation 22 allele frequencies with arcsine-square transformation using T-test, regularized T-test, and WRT test, we attempted to calculate a critical threshold by estimating the FDR of 10% [55, 56]. However, after calculating p-values for complete permutations of the different lines within linetype to better understand the null distribution, we concluded that this estimated FDR was underestimating the true false discovery rate. Therefore, using these same permutations, we calculated the FDR directly.

Direct calculations of FDR were performed by calculating FDR for each locus of the unpermuted data whose p-value was below 0.01 in accordance with the equation:

$$FDR=\frac{n\;False\;Positives}{n\;rejected\;Null\;Hypotheses}$$

This was implemented for each locus with:

$$FDR=\frac{\frac{n\;permuted\;loci\;significant\;at\;p}{35}}{n\;unpermuted\;loci\;significant\;at\;p}$$

Loci with nominal p-value < 0.05 were ordered by FDR score, the p-value was identified for the locus with the largest FDR below 0.01, and any p-values less than or equal to the p-value for this locus was treated as significant. The SNPs with FDR = 0.01 were then further grouped into “significant regions” by grouping any loci within 1mbp of another and separating groups whose closest SNPs are further than 1mbp.

### Divergence over time

To test for a difference in the number of loci showing a significant change in allele frequency between the HR and C lines from generations 22 to 61, we first conducted a paired T-test for the 4 C lines and separately for the 4 HR lines. These tests were based on eight data points for each linetype, i.e., the mean allele frequency for each line at a given locus at generation 22 and 61. The T-score for the C T-test was then subtracted from the T-score for the HR T-test, and the absolute value was taken. This was repeated for each locus, producing values for approximately 2 million loci (excluding where either the C or HR T-test failed for numerical reasons). These analyses were then repeated with all 35 permutations (as described above) to estimate the null distribution of the score based on ~2,000,000 * 35 = ~75,000,000 values. These scores were ordered to identify the 5^th^ percentile threshold for comparison with the distribution of the unpermuted results.

### “Strict” culling for biological and AF change analyses

Rather than attempt to focus on the genes of more than 100 regions for each of the different statistical tests, analyses of biological significance and comparisons of change in allele frequencies between generations 22 and 61 were done using a subset of the regions identified by FDR. WRT and regularized T-test first culled by removing regions containing only one significant locus, then culled such that only regions containing at least 20 significant loci or the lowest p-value among loci in the region was below 1.00E-04. Regions associated with the T-tests were culled in a similar manner as the WRT and RegT test, except the p-value cutoff used was 1.00E-06 due to naturally lower p-values. These culling methods should also serve to reduce the influence of sampling error, as it would be increasingly unlikely for sampling error to simultaneously underestimate among-line variance across multiple linked SNPs and lines. We will refer to these additional culling methods below as “strict” culling.

### Comparison of selection signatures in generations 22 and 61

Changes in allele frequencies from generation 22 to 61 were analyzed for each region identified by strict culling for generations 22 and 61. For regions significant at generation 22, each region and its included SNPs with nominal p<0.05 at generation 22 were matched with SNPs at generation 61. The allele frequencies of these SNPs were averaged for each line and generation and line graphs created (one for each line) with generation 22 AF on the left and generation 61 AF on the right. This was then repeated for regions significant at generation 61, except each region and its included SNPs with nominal p<0.05 at generation 61 were matched with SNPs at generation 22.

### Simulations to compare presumptive statistical power across generations

The available data from the two generations differ in multiple ways that might affect cross-generation comparisons of selection signatures. Each generation, each line is reduced to ~20 individuals when ~10 breeding pairs are formed. An ideal "sample" from a given generation would include all 20 of those breeding individuals. Instead, our sample from generation 22 was of ~10 males and 10 females per line that were sampled at random at the time of weaning (i.e., they were not the 20 breeding parents). In contrast, the mice from generation 61 were a semi-random sample of 10 males from each line (except nine from HR3 and one female that was unintentionally used from another line) [13].

For a pooled DNA sample, as for generation 22, a further ideal condition is for the sample of DNA from each mouse to be of equal volume and concentration through the extraction and pipetting steps prior to pooling. This would then result in each mouse’s alleles being represented in equal quantities in the pooled sequencing sample.

The next source of error is read depth, which is effectively a random sampling of alleles from the pooled sample. Our generation 22 samples were read at an average depth of 24X. Thus, the frequency of alternative nucleotide alleles for a given SNP locus was calculated by counting the number of alternative alleles, which was taken as anything other than the reference. Thus, not all of the 40 alleles (as one of two possibilities) contributed by the 20 mice could have been identified with a read depth of 24X, which acts as 24 samples taken with replacement.

The generation 61 data are from individual sequencing of 10 mice per line at an average read depth of 12X, with those results then used to predict the genotype for each SNP and mouse [13]. This should allow for the representation of nearly all alleles (N = 2 alleles x 10 mice). Originally, those data were analyzed as such via mixed models to detect selection signatures [13]. Here, to allow comparison with the pooled sequencing data from generation 22, we calculated allele frequencies as the number of alternative alleles divided by 2 times the number of mice (i.e., 20 or 18 for HR3), which should incorporate 19–20 unique alleles in equal proportion. Given that the data available from the two generations differ in multiple ways, we used simulations in an attempt to assess how this might affect our results.

For generation 22, simulations to elucidate possible sampling errors were performed such that alleles for 20 mice were sampled using a random binomial distribution assuming population allele frequencies of (0.05, 0.10, 0.15, …,0.90, and 0.95). Then an allele depth was randomly sampled from the actual quality data for the SNPs used in the generation 22 analyses and alleles were sampled from these simulated 20 mice (with replace) equal to this read depth. The allele frequency was then calculated as the number of alternative alleles (1) divided by the total read depth. This generated a distribution of allele frequencies given a particular starting AF for the population and was repeated 100,000 times for each starting population AF.

For generation 61, simulations were performed such that alleles for 10 mice were sampled using a random binomial distribution assuming population allele frequencies of (0.05, 0.10, 0.15, …,0.90, and 0.95). Then for each simulated mouse’s genotype, a genotype quality was randomly sampled from the actual quality data for the SNPs used in the generation 61 analyses. If the simulated genotype for the mouse was heterozygous, then the genotype quality would be used to generate a 0 or 1 with the probability of a 1 equaling that of the probability of a genotyping error. If a 1 was generated (thus an error occurred) the second allele for the mouse was replace with a copy of the first allele of the mouse. The allele frequency was then calculated as the number of alternative alleles (i.e., 1) for all ten mice divided by the total alleles (i.e., 20). This generated a distribution of allele frequencies given a particular starting AF for the population and was repeated 100,000 times for each starting population AF.

Power analyses were then done by sampling four AF values from the simulated AF values from an actual population AF of 0.4 for one linetype. Likewise, four AF values were sampled from the simulated AF values from an actual population AF of 0.6 for the other linetype. Sampled allele frequencies were transformed using an arcsine-squared transformation. A T-test (assuming unequal variance) was then conducted comparing these 8 sampled AF values. Note that this could not be done for RegT and WRT tests because it would require simulations of regional or genome-wide variance structure. These sampling and T-tests were repeated 10,000 times.

### Simulations comparing power with and without a biological constraint

We used simulations to begin to address whether a biological constraint on a trait under selection (e.g., wheel running) might affect (1) the ability to detect selection signatures at generations before (e.g., generation 22) versus long after (e.g., generation 61) selection limits were reached, (2) the consistency of those signatures across generations, and (3) the rate at which loci with different allelic effect sizes respond to selection. Our rationale for using a constraint model is explained in the Discussion. As a heuristic, some of the parameters in these simulations were chosen to approximate values observed in the selection experiment and help build a model of architecture for wheel running in the HR and control mice [57].

Running levels were calculated based on the general equation:

$$y=\mu +v_{g}+v_{e}$$

Where y is equal to the phenotype (wheel revolutions/day) of an individual mouse; μ is the "base" mean number of revolutions (held constant at the starting value set at generation 0); v_g_ is the variance contributed by genetic variation; and v_e_ is the variance contributed by environmental effects.

As a regression model, this equation is:

$$y=\mu +\beta_{1}X_{1}+\beta_{2}X_{2}$$

where the genetic variance is represented by β_1_X_1_ and the environmental variance is represented by β_1_X_2_. X_1_ represents the summed effect on wheel running of all alleles carried by the individual, where, to simulate a leptokurtic distribution [58–60], these alleles are coded as having variable allelic affects (specifically, ±0.4, ±0.8, ±1.6, ±3.2,… ±204.8) at frequencies inversely proportional to their effect size (specifically, 720 loci with effect ±0.4, 480 loci with effect ±0.8, … 8 loci with effect ±204.8) for a total of 2,096 loci, which approximates the number of haplotype blocks observed across all eight lines [13]. X_2_ provides the random element of the environmental variance and is determined by randomly sampling from a normal distribution with mean = 0 and SD = 1.

The equation we applied for these simulations is:

$$y=4,570+{1.3X}_{1}+{2,100X}_{2}$$

The values for β_1_ (1.3), β_2_ (2,100) and for the number of loci were determined in conjunction with one another to approximate realistic (in no particular order) (1) heritability of wheel running at the base generation being about 0.32 [3], (2) within-line coefficients of variation as being about 0.57 [15], and (3) realistic response to selection in the HR lines (i.e., achieving ~16,000 revolutions around generation 22) [3].

However, this equation does not adequately simulate seasonal variation see Appendix S5 in [3], so we applied an additional modifier:

$$y=S*(4,570+{1.3X}_{1}+{2,100X}_{2})$$

S is a constant that alternates cycles between 0.769 (summer), 1 (winter and fall), and 1.3 (winter). As generation time for the first 61 generations was consistently around 3 months, these constants can alternate with each generation. The mean of 4,570 (revolutions/day) was picked to approximate the empirically determined starting running levels at generation 0 [15].

Any running level calculated as below 100 was set to 100, which is approximately the lowest amount of running ever observed. The maximum wheel-running for unconstrained simulations was 50,000 revolutions, which is nearly twice as high as has ever been observed in actual measurements from the selection experiment [3, 61]. In practice, the highest running level produced by the unconstrained simulations was 38,875 (of 24,400 total individuals simulated over 61 generations for the HR lines).

For the starting population of any given line, two alleles were first assigned to each of the 2,096 independently segregating starting loci for 20 mice (based on the actual selection procedures: [15]) using a random binomial distribution with p = 0.5. For control lines, mice were paired, and alleles sampled from each of the pair to produce two male and two female offspring (to match the number of mice that are typically retained and wheel-tested in the selection experiment). The first of each sex for each family was then chosen to contribute to the next generation, which is functionally equivalent to the selection experiment, where breeders are chosen a random within family and sex for control lines. For HR lines, alleles were sampled from the parents for each of five males and five females (typical litter size is 10). Running distances were then calculated for all offspring, and the male and female with the highest running levels within each family were selected to breed for the subsequent generation (again, based on the actual selection procedure, which uses within-family selection). For both linetypes, siblings were barred from pairing (following the selection experiment). Simulations were run for 61 generations and alleles for all breeding pairs were saved at generation 0 and every 5 generations through 60, as well as generations 22 and 61. This was then repeated for 4 control lines and 4 HR lines.

We modeled the constraint on wheel running as a trait that itself can evolve. To obtain a realistic value for the constraint, we applied the same principles as for the wheel-running equation:

$$C=S*(10,000+{1.0X}_{C1}+{1,750X}_{C2})$$

C is the constraint to be applied to the mouse’s wheel running. S is the same seasonal multiplier used in the wheel-running equation, without which, higher running levels in winter become truncated. X_C1_ represents the genetic component of the constraint determined by (arbitrarily) 100 loci with effect sizes of ±1 (N = 48), ±4 (N = 24), ±11 (N = 12), ±36 (N = 8), ±101 (N = 5), and ±306 (N = 3). X_C1_ represents the environmental component, determined by sampling from a normal distribution with mean = 0 and SD = 1 (similarly to wheel running). These values result in a narrow-sense heritability of ~0.2. Despite targeting a wheel-running constraint of about 16,000 revolutions in the HR lines, the base constraint value is set to 10,000 because the alleles that increase constraint are favored by selection in the HR lines. Thus, a lower base value is needed for HR lines to stop responding to selection at about 16,000 revolutions. For the constrained simulation, if a mouse ran more than its determined constraint then its revolutions were treated as equal to the constraint itself before picking the breeders for the next generation.

These simulations were repeated 100 times (with 4 HR lines and 4 control lines in each simulation) assuming no constraint and 100 times with the constraint (see S2 File, for parameters). T-tests assuming unequal variance between the 4 control lines and the 4 HR lines were performed at each of these “saved” generations (0, 5, 10, etc.) for the allele frequencies at each locus, with an arcsine-squared transform [53]. Power was then calculated for each simulation at each saved generation by dividing the number of loci with p ≤ 0.05 by the total number of loci (N = 2,096). Power was also calculated separating loci by effect size (see below).

Standardized selection differentials were calculated following Careau et al. [3], by subtracting from the mean running for each sex and family the running level of the bred individual from that litter and dividing the difference by the standard deviation of the sex for that litter. Relative power under the constrained and unconstrained models was calculated using unpaired T-tests (unequal variance) on the previously described power calculations for each simulation and for each saved generation. Relative power across generations was also calculated using unpaired T-tests (unequal variance), separately for constrained and unconstrained simulations. Relative consistency in detected selection signatures was calculated by first identifying the specific significant loci (at a nominal α = 0.05) at generations 22 and 61 in each simulation. Then, the percentage of loci found significant at generation 22 that remained significant at generation 61 was calculated. Unpaired T-tests (unequal variance) were performed comparing these percentages for the constrained simulations versus the unconstrained simulations. Lastly, ability to detect loci with different effect sizes was compared using a T-test (unequal variance) of the number of significant loci (p ≤ 0.05) identified for each effect size and each simulation for generation 22 constrained vs unconstrained models, generation 61 constrained vs unconstrained models, constrained generation 22 vs generation 61, and unconstrained generation 22 vs generation 61. For all graphs and estimates that required the calculation of a mean value, missing values were excluded from the calculations. For example, if a p-value could not be calculated for a given locus due to fixation across all lines for the same allele, then this locus would be excluded from the power analyses.

Analyses were performed again implementing possible sampling error calculated by the simulations to compare statistical power, as described in the previous section "Simulations to Compare Presumptive Statistical Power Across Generations". This was implemented by taking the actual allele frequency for each line at generations 22 and 61 in the simulations using the constraint model. These allele frequencies were then replaced with an allele frequency sampled from the results (rounded to the nearest 0.05) of the population allele frequency of the sampling error simulations (i.e., 0.05, 0.10, 0.15… 0.95). For example, if the allele frequency for a given line at generation 22 (constraint model) was 0.25, then this 0.25 would be replaced by a randomly sampled estimated allele frequency from the sampling error simulations (generation 22) where 0.25 was the actual population allele frequency. Generation 61 allele frequencies were similarly replaced using the results of the generation 61 sampling error simulations.

### Ethics statement

The selection experiment has been carried out in strict accordance with the approval from the Institutional Animal Care and Use Committee (IACUC) at two different institutions and under multiple protocol number. All experiments have been conducted to minimize distress to the animals. Any injuries or illness were treated in accordance with veterinarian recommendations. The present manuscript uses only published sequence data and new sequence data from historical tissue samples.

## Results

### Basic characteristics of genetic variation

**Fig 1:**
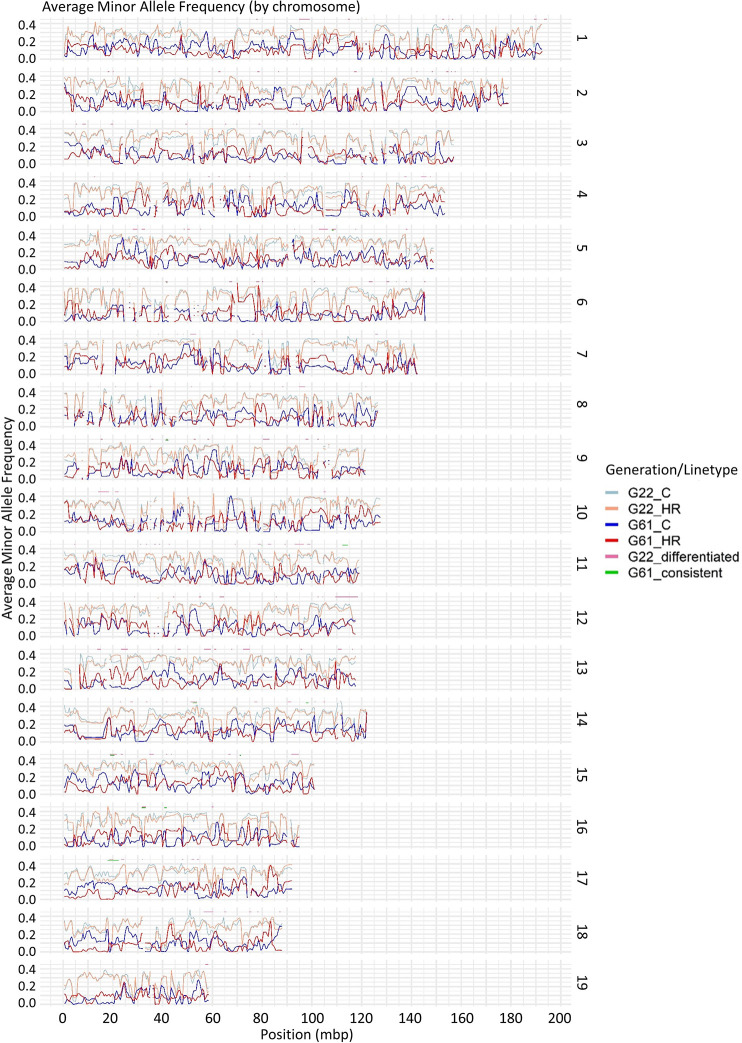
Average minor allele frequencies. Average minor allele frequencies for generation 22 control lines, generation 22 HR lines, generation 61 control lines, and generation 61 HR lines by chromosome (numbered on the right). Regions identified as differentiated at generation 22 are indicated with an orange line above each chromosome’s graph (regions smaller than 50 kbp are omitted). Regions identified as consistently differentiated at generation 61 (Hillis et al., 2020) are indicated similarly with a green line.

The number of variable loci used in the present study includes 4,446,523 for generation 22 and 5,932,148 for generation 61. Generation 61 data had an average read depth of 12X per mouse for 10 mice in each of the 8 lines, producing an average read depth of over 100 per line for detection of many more variable SNPs in each line. The overlap of base positions between generations 22 and 61 was 2,045,546 SNPs. As expected, minor allele frequency (MAF), generally decreases for both HR and C lines between generations 22 and 61 (Fig 1). MAF values for HR and C lines are generally similar at generation 22; however, these diverge for many regions by generation 61.

### Differentiated SNPs and chromosomal regions

**Fig 2:**
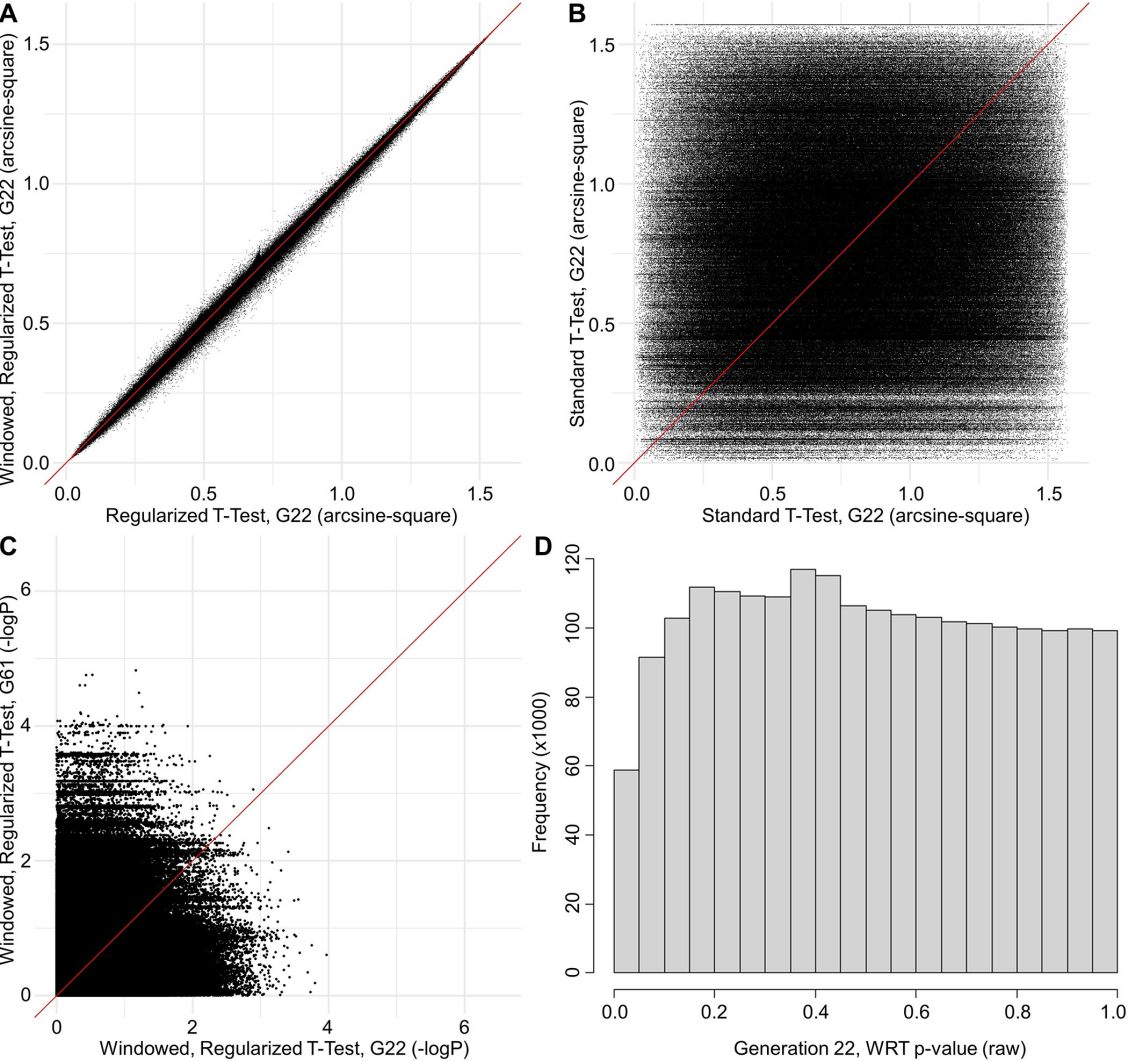
P-value comparisons between generations. Scatterplot comparisons of the generations 22 and 61 p-values with Pearson’s correlation: (A) Generation 22 regularized T-test vs generation 22 WRT test (cor = 0.9997). (B) Generation 22 WRT test vs generation 61 WRT test (cor = 0.0909). (C) Generation 22 WRT test vs generation 61 WRT test (cor = 0.1156). (D) Distribution of raw p-values (generation 22).

**Fig 3:**
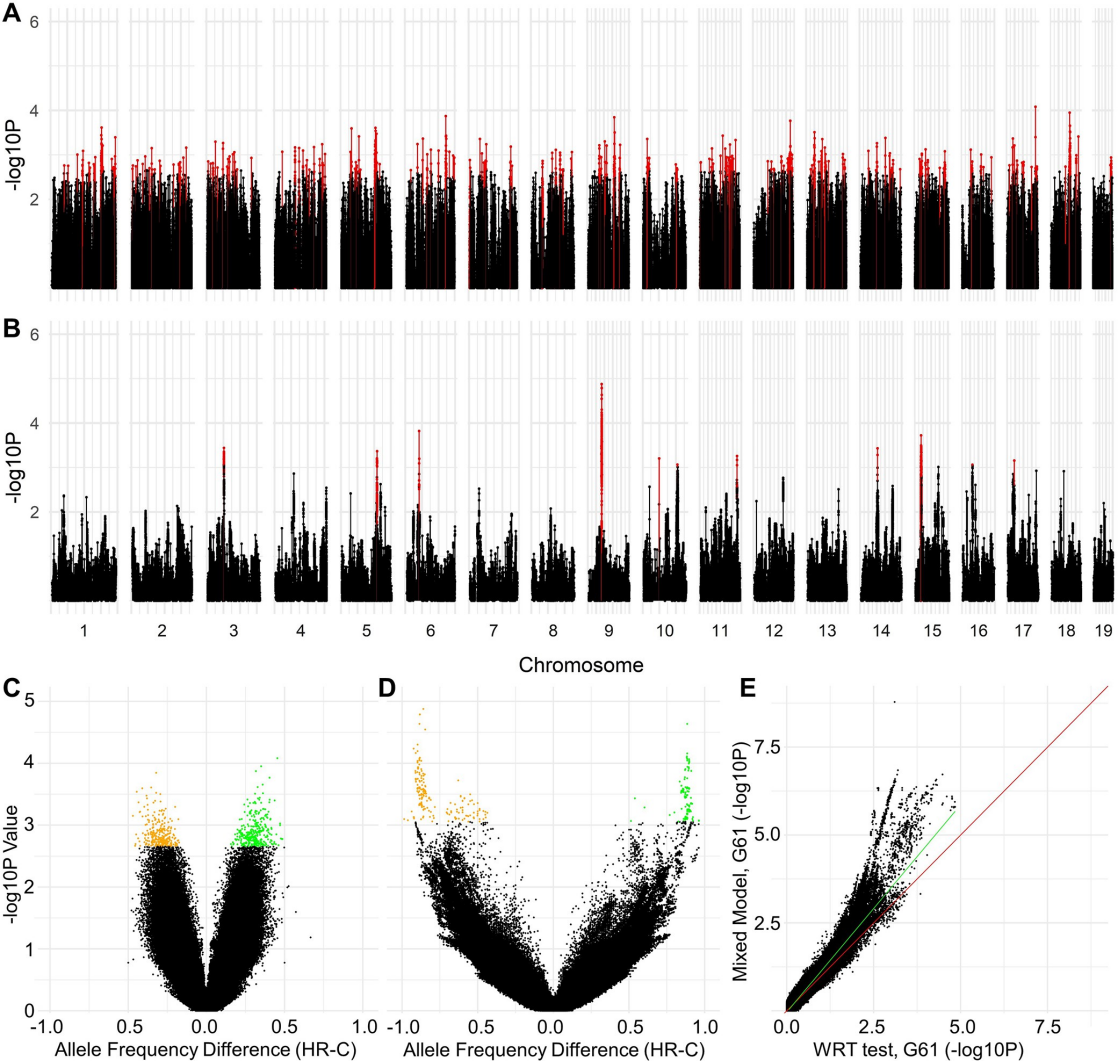
Manhattan plots and volcano plots of differentiation analyses. Manhattan plots for results from (A) the generation 22 WRT test (shared loci), (B) the generation 61 (pooled) WRT test (shared loci). The red peaks indicates those that exceeded critical threshold (FDR = 0.01) for that individual test. Volcano plot including -logP vs HR allele frequency minus C allele frequency for (C) generation 22 and (D) Generation 61 (orange points indicate HR AF > C AF; green points indicate HR AF < C AF). (E) Scatterplot comparing the -log p-values of the generation 61 mixed model analyses (individual mouse) with -log p-values produced when these same data are treated as pooled sequencing allele frequencies and analyzed with WRT test. Red line has intercept = 0 and slope = 1. Green line represents the least squares regression line.

For analyses containing all generation 22 loci (N = 4,446,523), WRT identified 1,184 differentiated loci based on 0.01 FDR (Table 1). These loci fall into 258 unique regions (separated by at least 1 million base pairs). At generation 61, 1,449 loci were identified as differentiated based on 0.01 FDR. Although identifying similar numbers of loci as the generation 22 analyses, P-values for individual SNPs for generations 22 and 61 show little similarity (Fig 2B and 2C), with arcsine-square transform Pearson’s r = 0.116. Ultimately, the SNPs identified at generations 22 and 61 were largely different. Moreover, the SNPs identified at generation 61 clustered into only 11 unique regions, as compared with the 258 regions for generation 22 (Fig 3A and 3B).

**Table 1:** WRT results.

Data	Total Loci	FDR 0.01 (-logP)	Significant SNPs	All Regions	Regions after strict culling
Gen22AF	2,045,546	2.66	630	187	6
Gen61AF	2,045,546	3.06	1,285	11	4
Gen22AF	4,446,523	2.62	1,184	258	13
Gen61AF	5,932,148	3.23	1,449	11	5

Number of SNPs listed represents those that are statistically significant based on a False Discovery Rate of 1% using permutations. Analyses with 2,045,546 loci incorporate only loci which are shared between generations 22 and 61. Regions distinguished by being separated from the next closest significant locus by more than 1 million bp. Additional regions remaining after additional culling methods have either 20 significant loci or at least 2 significant loci with one having a p-value <1.00E-04.

Given such notable differences between the SNPs and regions implicated by generation 22 and 61 analyses (Table 2), analyses were repeated focusing only on the loci found in both data sets (N = 2,045,546). With fewer loci being analyzed, fewer significant SNPs were identified at FDR = 0.01, as well as fewer regions for all analyses except for WRT with generation 61. The total peaks identified when using only the shared SNPs includes 187 and 11 regions for generations 22 and 61, respectively.

**Table 2:** Genomic regions identified as differentiated under “strict” culling.

G22 Region	G22 WRT	G61 Region	G61 WRT	Chr	minPOS	maxPOS	Size	Loci	Shared Loci
1	x			1	152,318,219	153,239,876	921,658	40	25
2	x			2	78,021,909	78,974,325	952,417	3	0
		1	x	3	51,199,110	51,602,693	403,584	124	65
3	x			5	32,384,612	32,975,871	591,260	32	4
4	x			5	102,846,390	106,315,986	3,469,597	63	37
		**2**	**x**	**6**	**40,933,658**	**41,748,676**	**815,019**	**5**	**1**
5	x			6	122,815,876	124,446,843	1,630,968	43	20
		**3**	**x**	**9**	**41,413,436**	**42,478,817**	**1,065,382**	**1,277**	**647**
6	x			9	80,349,989	82,894,555	2,544,567	27	20
7	x			10	14,067,617	18,376,599	4,308,983	25	7
8	x			10	20,890,526	21,419,406	528,881	33	4
		4	x	10	104,966,751	105,529,701	562,951	2	0
9	x			13	46,088,694	46,866,721	778,028	33	13
**10**	**x**	** **	** **	**14**	**52,115,206**	**53,776,455**	**1,661,250**	**42**	**7**
11	x			14	77,333,032	78,080,942	747,911	4	3
		**5**	**x**	**15**	**19,245,017**	**20,197,326**	**952,310**	**27**	**17**
12	x			18	57,707,454	60,118,834	2,411,381	128	81
13	x			18	78,018,740	78,504,680	485,941	4	4

A test is deemed to have produced a differentiated region if the region contains at least 20 SNPs significant at FDR = 0.01 or at least 2 SNPs significant at FDR = 0.01 and at least one SNP with p-value < 1.0E-04 (See Methods: Determination of Selection Signatures). “Loci” listed are those significant at FDR = 0.01 (Table 1), the counts themselves match the number of differentiated loci identified by the statistical test with the most such loci. “Shared Loci” are the number of loci listed in the “Loci” column that are also shared between both generations.

**Bolded** loci match “consistent” regions identified by Hillis et al. [13]

### Regions after “strict” culling

Using all available SNPs for generation 22, after applying “strict” culling (see Methods), the remaining regions were reduced to 13. All of the regions implicated by these analyses included or were near genes with intuitive implications for running behavior (see Discussion). For generation 61, strict culling reduced the total peaks to only 5 unique regions.

Despite the HR lines reaching selection limits around generation 22 or shortly thereafter [3], the most differentiated 13 regions (Table 2) have little fixation. Of the SNPs in these regions (N = 79,198), only about 8.78% are fixed in the HR lines, which is not significantly different from the 9.21% fixed in the control lines (unequal variance t-test comparing % fixed in the 4 HR versus 4 C lines: p-value = 0.4322). If we repeat this fixation comparison for the loci shared between generations 22 and 61 (N = 42,745), 1.62% are fixed in the HR lines, which is still not significantly different from the 1.58% fixed in the control lines (p-value = 0.6129).

### Comparison of selection signatures at generation 61 for individual vs. pooled sequencing data

Originally, the generation 61 individual mouse data were analyzed using mixed models [13]. We compared the previously published p-values from those analyses with the p-values produced after pooling data by line and analyzing by the WRT test (Fig 3E). The mixed model analyses produced lower p-values in general, as would be expected due to loss of power with pooling [52], with the difference being greater for lower p-values. As a result, fewer SNP loci and hence fewer chromosomal regions were identified as significantly differentiated between the HR and C lines with pooled data. Of the total regions detected with FDR = 0.01, 7 were identified at generation 22 that matched the 13 “consistent” regions identified with the mixed model analyses [13]. The 6 consistent regions that were not identified by analyses of the pooled data tended to have relatively large p-values for individual SNP loci or cover a narrower area of the genome, as compared with the other 7 consistent regions.

### Divergence over time

The 5th percentile threshold for the difference in T-scores determined by permutations was 5.139787. About 6.44% of the T-scores for unpermuted data were larger than this. This difference of 6.44–5% indicates that ~1.44% of our values for the real data (approximately 28,470 SNPs) may be considered nominally statistically significant for α = 0.05. This result provides statistical support for our claim that the selection signatures differ between generations 22 and 61 (see Discussion). Defining a region as containing at least 20 significant SNPs with no adjacent SNPs separated by more than 1mbp, and considering the 1,400 most significant SNPs, they cluster into 14 regions on 13 chromosomes (Table 3).

**Table 3:** Genomic regions of divergent evolution.

Chr	minPOS	maxPOS	Loci	Median_T	Highest_T	T_position	g22	g61^a^
1	152,255,010	153,208,066	44	48.9	94.9	152,795,939	WRT	
1	156,267,494	156,908,946	21	48.5	115.5	156,699,891		
2	153,709,397	157,032,971	20	46.0	102.8	154,908,418		1 Test
3	45,831,668	52,497,670	49	51.0	139.9	51,543,977		2 Tests
4	89,020,582	90,615,570	26	52.5	103.3	90,007,699		1 Test
5	107,675,741	111,271,760	30	55.2	232.2	109,810,077		Consistent
9	41,416,364	42,248,169	31	47.3	99.8	41,533,058		Consistent
10	101,702,890	105,671,151	154	47.4	177.0	103,108,543		2 Tests
12	109,050,203	110,779,901	28	46.6	109.2	109,228,613		
14	96,560,689	98,613,561	76	49.5	212.2	97,831,005		Consistent
15	18,635,736	20,608,793	39	51.4	208.5	19,984,048		Consistent
16	45,132,582	47,948,007	23	46.5	163.9	45,158,346		
18	69,603,969	74,277,731	62	46.7	147.5	73,016,958		
19	35,121,427	35,736,790	40	48.7	250.1	35,699,388		

^a^Tests here are the three tests used by Hillis et al. 2020 (local maxima, haplotype, and FixedHR/PolyC) [13]. “Consistent” is the term used to describe regions identified by all three tests.

### Simulations to compare presumptive statistical power across generations

**Fig 4:**
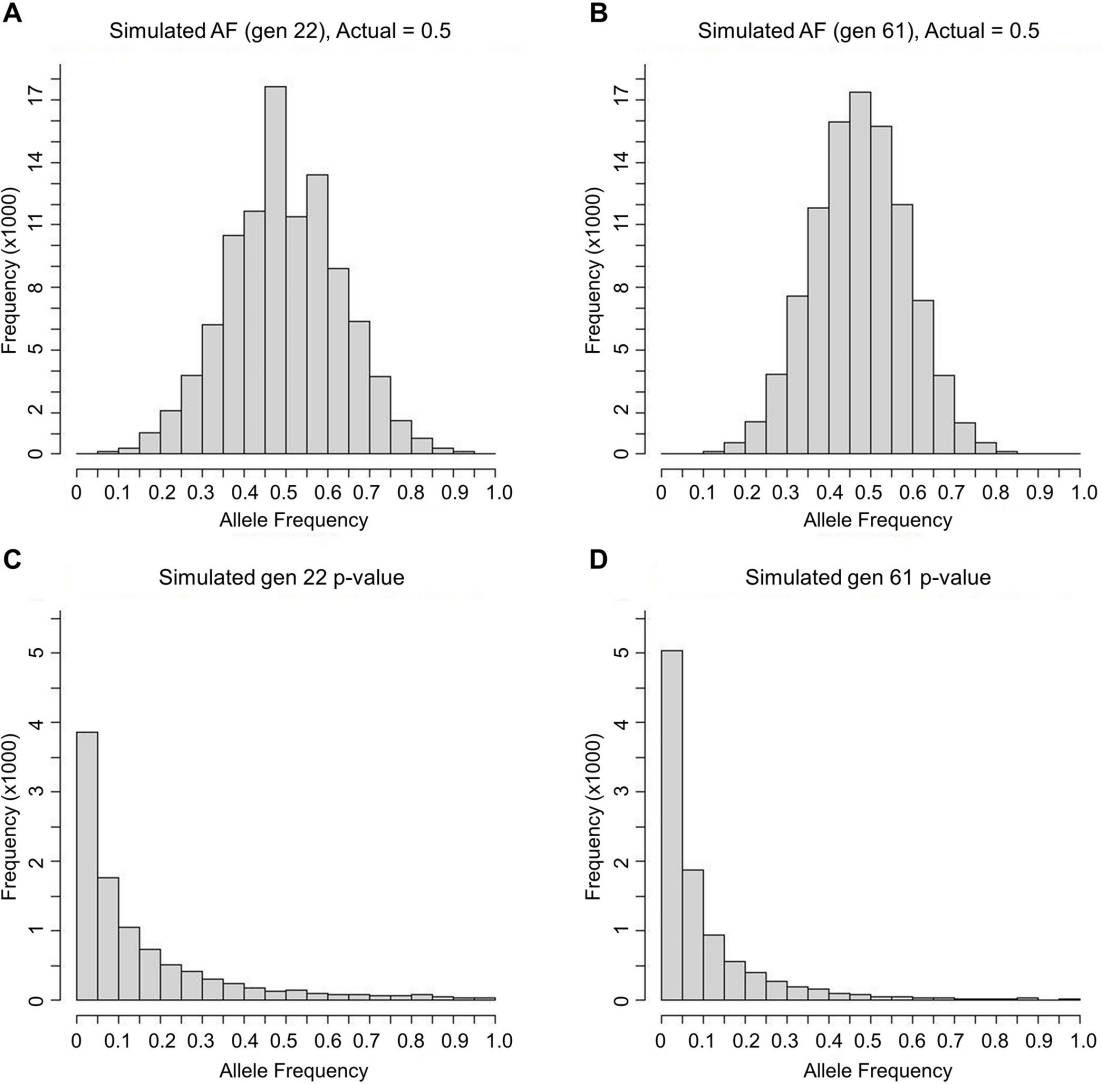
Variance and power simulation results. Simulations for a population allele frequency of 0.5 for (A) generation 22 and (B) generation 61. See Methods for details. Values shown are allele frequencies for each of 100,000 simulated data sets for a single line. Methodological differences in the sampling of mice and sequencing procedures for the two generations result in greater sampling error for generation 22 (i.e., larger SD). Note that binning is done such that loci that fall on a break (e.g., 0.05) are grouped into the lower bin (e.g., 0 to 0.05). Similar simulations were then conducted to create data sets for use in estimating statistical power for detecting selection signatures for generations 22 and 61. (C) Distribution of p-values for simulated allele frequencies of 0.4 versus 0.6, for generation 22. Power for α = 0.05 is 0.3864. (D) Distribution of p-values for simulated allele frequencies of 0.4 versus 0.6, for generation 61. Power for α = 0.05 is 0.5031.

Simulations were conducted to gauge how much the allele frequencies determined through sequencing reflect allele frequencies of the actual populations at generations 22 and 61. Generation 22 allele frequencies have greater variance from the actual population AF than generation 61 (see Fig 4A and 4B for an example of the 0.5 population AF distribution). The greater error variance in generation 22 is associated with reduced statistical power of 0.3864 versus 0.5031 for generation 61 when comparing simulated allele frequencies of 0.4 and 0.6 (Fig 4C and 4D).

### Simulations comparing power with and without a biological constraint

**Fig 5:**
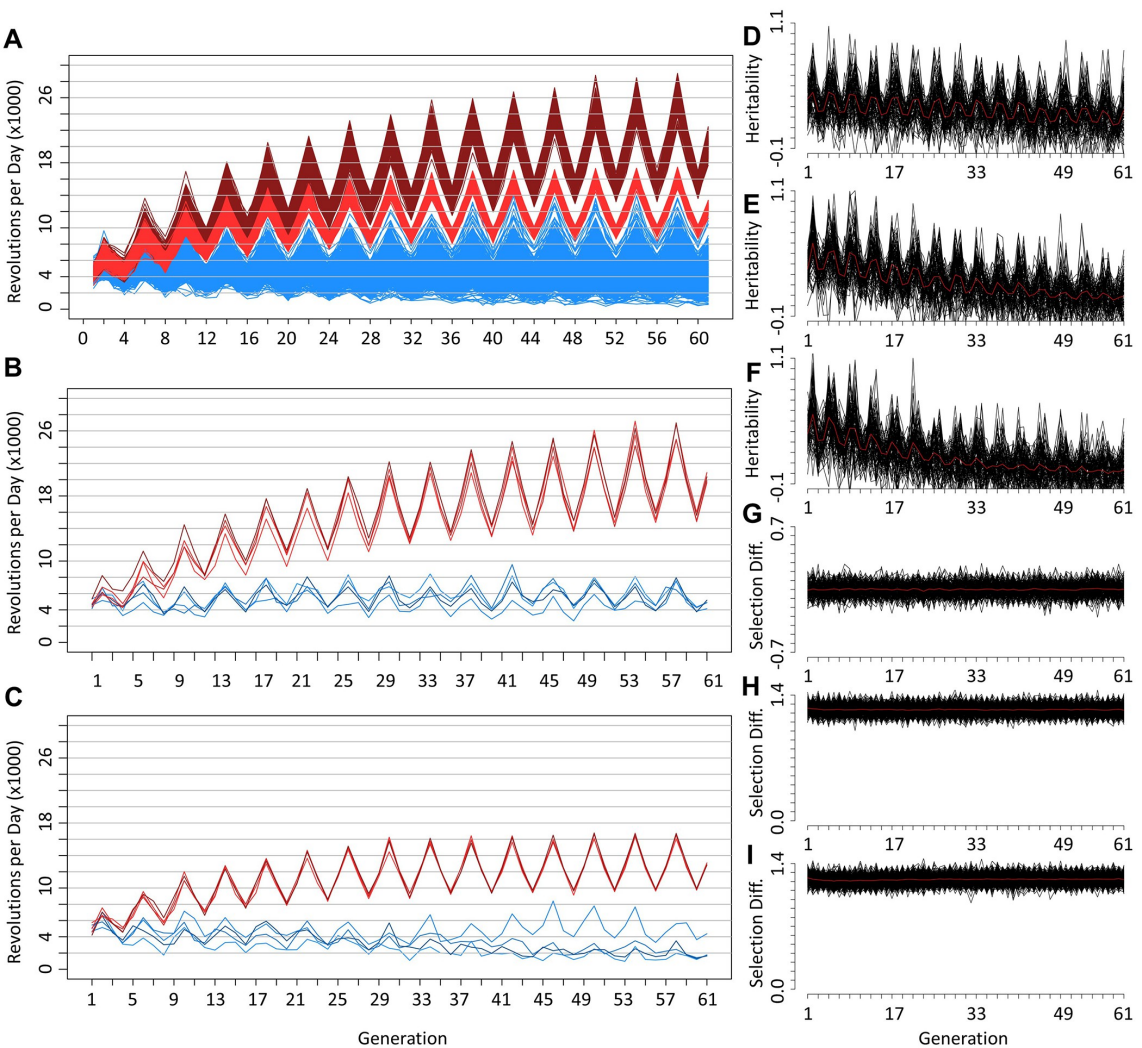
Power simulations considering a constraint. Simulated running levels for (A) mean running of 4 non-selected control lines (blue) for 200 simulations compared with mean running of 4 HR lines under 100 unconstrained simulations (dark red) and 100 constrained simulations (light red). (B) Individual HR and control lines single unconstrained simulation. (C) Individual HR and control lines single constrained simulation. Black lines show the mean narrow-sense heritability for four lines within each linetype for (D) 100 control line simulations (arbitrarily the first 50 from each model), (E) 100 simulations for HR lines with unconstrained running, and (F) 100 simulations for HR lines with constrained running (16,000 revolutions). Standardized selection differentials (calculated within family and sex) from simulations for (G) control lines, (H) HR lines without a constraint, and (I) HR lines with a constraint. The red line represents the mean of all heritability and selection differentials (N = 100). Note different axes for panel G versus H and I.

Simulations were performed modeling response to selection assuming either a constraint with a base of 10,000 revolutions per day and the capacity to evolve to about 17,000 (in the winter) or no such constraint (see Methods). For both constrained and unconstrained simulations, wheel running for HR and control lines diverge recognizably at least by generation 6 (Fig 5A), consistent with the selection experiment. The replicate HR lines for unconstrained and constrained models appear fairly similar for earlier generations (Fig 5B and 5C), presumably because mice are not widely achieving constrained running levels. As expected, the among-line variation for control lines increases gradually across generations. For the HR lines, among-line variance does not increase to a noticeable extent and potentially even diminishes by later generations, a result that is also consistent with the selection experiment [49].

The calculated heritability (slope of the regression of offspring [generation 1] on midparent [generation 0]) for all 200 simulations for control lines indicate that our parameters resulted in a narrow-sense heritability of about 0.3621 (N = 8,000 families). For individual lines, the estimated heritability for successive generations was highly variable, as would be expected with such small sample sizes (10 families/line). However, the means clearly indicate a slow loss of heritability in the control lines and a more rapid loss in the HR lines, although values never go to zero (Fig 5E and 5F), consistent with the selection experiment [3].

The standardized selection differentials (calculated within family and sex) for the unconstrained model remained very consistently around 0 for the control lines (Fig 5G) and 1.2–1.3 for the HR lines (Fig 5H and 5I). However, the constrained selection differential is on average 0.016 below the unconstrained differential. Although slight, this difference remains consistent across nearly all generations (graph not shown). In the actual selection experiment, selection differentials declined across generations [3].

Under both models, Type I error rate for α = 0.05 when comparing allele frequencies of HR with C lines was deflated at generation 0, regardless of the effect size for the locus. Type I error ranged from 0.0313 to 0.0420 with no preference for any effect size (S1 Table). This relatively low power when comparing the HR and control lines (when the line itself is the experimental unit) has been documented previously with simulations for both genetic data and phenotypes [13, 62].

As expected, power to detect differentiation between the HR and C lines increased across generations, but never exceeded 0.057 for any generation for either model. Comparing models at each generation indicates that power is significantly higher under the unconstrained model by generation 15, although the difference is trivial (0.0015 with P = 0.0127) (Table 4). This differential in power increased through generation 50, when it reached 0.0055 (P = 2.63E-15), before beginning to diminish with later generations. Although the information in Table 4 does not tell us about the power to detect loci based on effect size (see numbered list below), it does establish that we expect more total selection signatures at generation 61 than 22 (see Discussion).

**Table 4:** Simulated statistical power for constrained and unconstrained models.

Generation	Unconstrained power	P-value for Unc. Comparing present generation to previous generation^a^	Constrained power	P-value for C. Comparing present generation to previous generation^a^	P-value for Constrained vs Unconstrained^b^
0	0.0408^c^	NA	0.0409^c^	NA	0.8436
5	0.0417	0.1192	0.0423	0.0365	0.3688
10	0.0429	0.0379	0.0429	0.2685	0.9817
15	0.0447	0.0060	0.0432	0.6693	0.0127
20	0.0456	0.1272	0.0425	0.1891	2.69E-07
22	0.0461	0.4333	0.0425	0.9470	2.31E-07
25	0.0465	0.5290	0.0434	0.1721	1.58E-06
30	0.0484	0.0042	0.0447	0.0374	4.61E-08
35	0.0503	0.0041	0.0463	0.0155	4.51E-09
40	0.0527	0.0006	0.0476	0.0423	5.31E-13
45	0.0544	0.0160	0.0497	0.0009	6.42E-11
50	0.0557	0.0584	0.0502	0.4380	2.63E-15
55	0.0562	0.4050	0.0510	0.2169	2.40E-13
60	0.0565	0.7307	0.0512	0.8162	3.70E-13
61	0.0563	0.8387	0.0512	0.9147	6.27E-13

^a^ These p-values are calculated using a T-test assuming unequal variance comparing the power of the generation for that line to the previously listed generation (e.g., unconstrained power at generation 5 compared to generation 0 has a p-value of 0.1317).

^b^ P-value from a T-test (unequal variance) comparing the power of the 100 unconstrained simulations to the 100 constrained simulations

^c^ Type I error rate.

The average Pearson correlation between p-values across 2,096 loci for generation 22 and 61 for the constrained model (r = 0.3843) was not statistically different from that for the unconstrained model (r = 0.3889: total N = 200, unpaired-T = -1.6938, P = 0.0919). In the unconstrained model, 33.3% of the loci significantly differentiated at generation 22 (α = 0.05) were still differentiated at generation 61, versus 31.5% under the constrained model (unpaired-T of percentage of loci consistently different for generations 22 and 61 at p ≤ 0.05 for 200 simulations = -2.4269, P = 0.01614). This consistency of about 1/3 is more than 3 times greater than for the real data (9.12% for T-tests), which mirrors the drop in the correlation of p-values between generations 22 and 61 (Fig 2B, r = 0.0898). Incorporation of sampling error into the constrained model lowered the correlation between p-values to 0.2695 and the proportion of loci significant at generation 22 still significant at generation 61 to 22.7%.

Comparisons of power to detect differentiation between the HR and C lines in relation to effect size of locus and generation under two simulation models (S2 Table) indicates:

1. power increased with effect size, as expected;
2. at generation 22, power was greater in the unconstrained model for loci with effect sizes 25.6 or above;
3. at generation 61, power was greater under the unconstrained model for loci with effect sizes 12.8 and above (excluding the largest effect size, 204.8);
4. under both models, power was consistently greater at generation 61, except for the largest effect size, where the power is reversed.

### Comparison of selection signatures in generations 22 and 61 for pooled data

**Fig 6:**
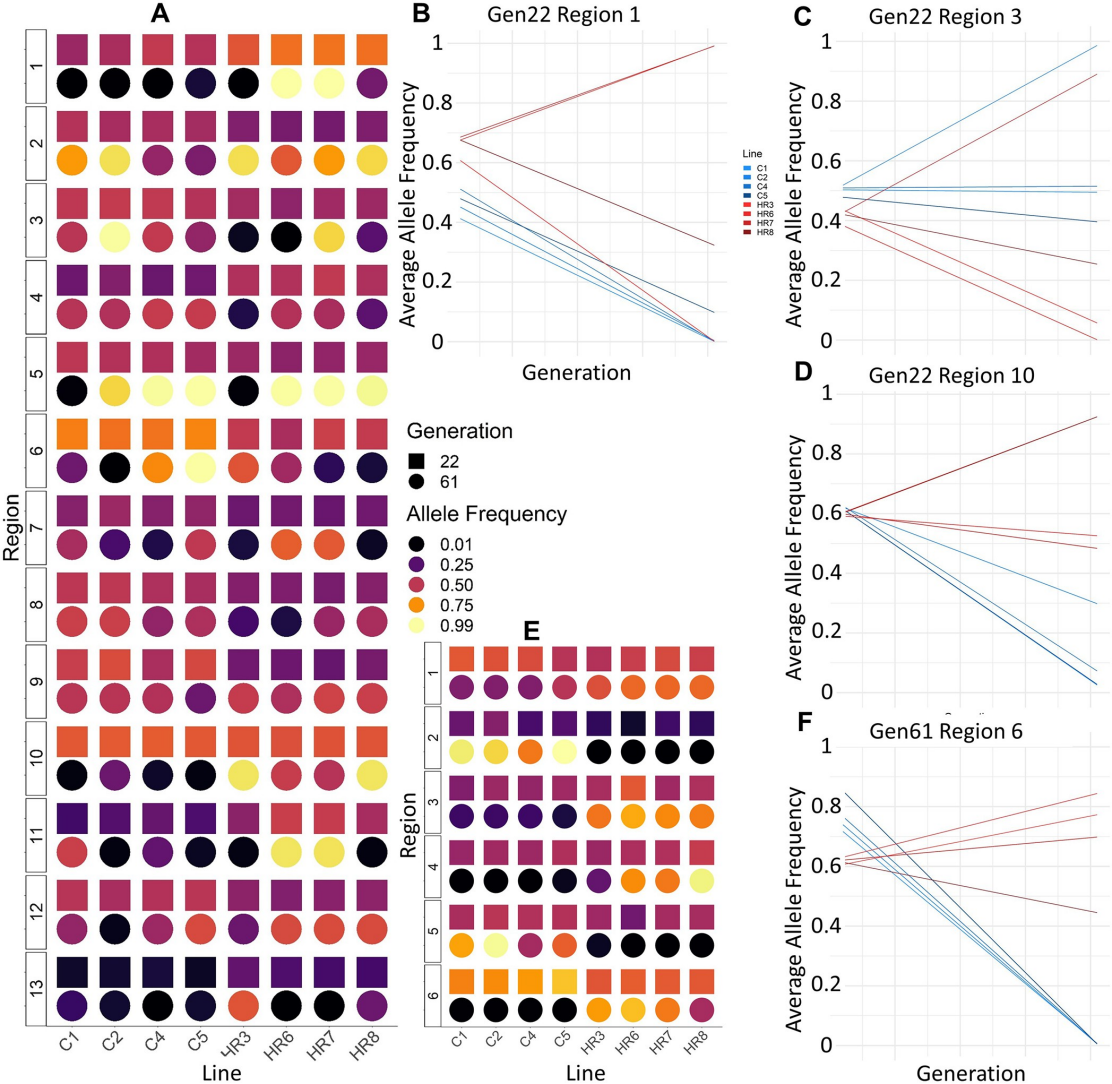
Power simulations considering a constraint. (A) Allele frequencies of regions identified as significant (via strict culling) at generation 22 (Table 2) (excepting region 18, for which no loci were available in the generation 61 data). Line plot examples are provided for generation 22 (B) region 1, (C) region3, and (D) region 10, with generation 22 on left and generation 61 on right. (E) Allele frequencies of regions identified as significant at generation 61. (F) Line plot example for generation 61 region 6. SNPs included have a nominal p<0.05 at generation 22 and any SNPs at generation 61 which matched the generation 22 SNPs (shared loci, see Table 1).

When the average allele frequencies of SNPs within regions identified by strict culling at generation 22 (this study) are compared to the average AF of those loci at generation 61, an increase in among-line variance is apparent for generation 61, within both the HR and C linetypes (Fig 6A). All else being equal, this increase in among-replicate variance should lower the statistical power to detect differentiation between the HR and C linetypes. In agreement with this expectation, most of these strict regions at generation 22 (Table 2) are no longer significantly differentiated at generation 61 (Table 2 and Fig 6A). However, several of the 5 strictly culled regions at generation 61 also show some evidence of differentiation at generation 22 (Fig 6E). Although strict culling methods exclude regions identified with generation 61 AF analyses, regions implicated in generation 22 with FDR = 0.01 culling alone do have considerable overlap with some of these 5 regions identified at generation 61. Generation 61 regions 3, 4, and 5 (Table 2) were significant at FDR = 0.01 for all three analyses at generation 22 (S3 Table).

For some of the regions identified as significant at generation 22, differentiation may have been lost by generation 61 as result of a single line diverging from the others (for example, line 3 in region 1 or line 7 in region 3 [Fig 6B and 6C]). In general, mean differences at generation 22 are much smaller than at 61, but also with much less among-line variance. A particular example of this includes region 10 (Fig 6D), which is the only region identified at generation 22 (after strict culling) to continue to be detected as differentiated at generation 61 (see Discussion).

### Possible biological function of generation 22 differentiated regions

A total of 79 genes (including predicted genes and miRNA) were identified using “strict” culling of generation 22 regions. These were insufficient for powerful ontology tests and so regions containing at least five differentiated loci at FDR <0.01 were included, bringing the total number of included genes to 345 (S4 Table). Of these 345 differentiated genes, 285 were recognized by the Panther database (used by the Gene Ontology Resource) and used for identifying potential biological function. Those not recognized were generally miRNA, predicted, or olfactory genes.

GO Biological Functions implicate antifungal innate immune response, sensory perception of smell, and embryonic skeletal system morphogenesis. The antifungal enrichment appears to be the result of a group of C-type lectin genes found on chromosome 6 (chr6:122,815,876–124,446,843). It may not be a coincidence that this region also includes a group of vomeronasal genes contributing to the sensory perception of smell term. The genes that implicate the embryonic skeletal morphogenesis term include a cluster of *Hoxb* genes found on chromosome 11 (chr11:93,129,916–96,570,699).

A few genes specifically from the 79 found in the “strict” regions merit mention for their relevance to the running phenotype, including *Cited2* (adrenal cortex formation), *Rbm24* (positive regulation of skeletal muscle fiber differentiation), and *Dspp* (negative regulation of bone development).

## Discussion

### Overview

Previously, whole-genome sequence data for individual mice at generation 61 of the High-Runner mouse selection experiment were used to identify genomic regions differentiated between HR and control lines. Thirteen of these were termed "consistent" because they appeared with three different analytical methods [13]. These 13 regions contained genes associated with known phenotypic differences between the HR and control lines and intuitive associations with running ability and/or motivation/reward systems. However, given that the HR lines had begun to reach selection limits around generations 17–27, depending on line and sex [3], tens of additional generations, with continuing random genetic drift, could have obscured many selection signatures. Therefore, in the present study, we analyzed allele frequencies for the lines sampled at generation 22, based on DNA pooled by line. These analyses of generation 22 identify hundreds of genomic regions differentiated between the HR and C lines (FDR = 0.01), despite using pooled sequence data rather than sequences for individual mice [52]. We then reanalyzed the data from generation 61 as allele frequencies by line, to mimic the data available for generation 22, and found that the regions identified as differentiated at generation 61 are, at best, weakly differentiated at generation 22. Nevertheless, both generations’ differentiated regions contain genes that make biological sense for wheel-running behavior. Below, we discuss (1) implications of the differences in data type between generations 22 and 61, (2) possible statistical and biological explanations for the differences in identified regions, and (3) genes and biological systems highlighted by the genomic regions identified by generation 22 analyses (after strict culling).

### Differences in selection signatures at generations 22 and 61

We expected estimates of selection signatures to be similar at generations 22 and 61, based on the fact that the HR lines had mostly reached selection limits by generation 22 [3], such that the most biologically important loci would have gone to fixation or at least reached equilibria across most or all of the HR lines. In agreement with this expectation, of the 13 "consistent" regions identified by Hillis et al. [13] for generation 61 (using individual mouse data), 8 were still identified by at least one of the tests (FDR = 0.01) using the generation 61 genotypes pooled into allele frequencies per line. Generation 22 analyses of pooled sequence data identified 7 of the 13 consistent regions (although several of these regions were only detected by a few SNPs: S3 Table). Interestingly, the consistent region on chromosome 14 was more strongly detected at generation 22 than at generation 61 using pooled sequence analyses (Table 2).

On the other hand, the strongest selection signatures observed at generation 61 with the data treated as pooled sequences are not among the strongest ones observed at generation 22 (based on number of SNPs detected and their p-values), despite continued selection on the HR lines. For example, region 1 of the generation 61 “strict” culling pooled analyses (chr3:51,199,110–51,602,693) included 124 SNPs (Table 2), and whereas generation 22 analyses did not detect any of these loci as significant. Another example, region 3 of the generation 61 pooled analyses (chr9:41,413,436–42,478,817) included 1,277 SNPs (FDR = 0.01), but none of the 647 shared loci were identified by in the generation 22 analyses (S3 Table). When directly comparing SNPs differentiated at FDR = 0.01, we see only a single SNP of overlap for the WRT test.

In addition to the differences in individual SNP results, a 17-fold greater number of regions was identified by generation 22 analyses than generation 61 pooled analyses at FDR = 0.01 (Table 1). This ratio applies to all statistical tests and the complete SNP analyses for each generation, as well as the analyses of SNPs shared by the two generations. Moreover, the SNPs identified at generation 61 were clustered into far fewer regions (Table 1). Broadly, this difference in numbers of selection signatures have at least two possible explanations, which are not mutually exclusive: (1) differences in data type, quality, and quantity; (2) biological differences between generations 22 and 61.

#### Differences in data type, quality, quantity, and sampling error

Our power to detect differentiation in allele frequencies should have been lower for generation 22 than for generation 61 (Fig 4C and 4D). As also noted in the Methods, the estimates for SNP allele frequencies per line at generation 61 were based on ~10 mice/line sampled and an 12X average read depth per mouse, yielding a total of 5,932,148 variable SNP loci [13]. For generation 22, pooled sequencing was done with ~20 mice/line and an average read depth of 24X, yielding 4,446,523 variable SNPs (Table 1). Generally, with an average read depth of 12X per mouse, both alleles will be represented for each mouse (i.e., 20 alleles per line) for generation 61 allele frequencies. However, with 24X average read depth for generation 22, simulations involving sampling alleles with replacement show that generation 22 is prone to vary more from the actual population allele frequency (Fig 4A and 4B). Thus, the much greater number of differentiated SNPs and chromosomal regions detected at generation 22 would not appear to be simply a function of greater statistical power versus generation 61. Thus, we now consider possible biological explanations.

#### Biological differences

One way to highlight the differences in selection signatures detected at generations 22 and 61 is to note that of the differentiated regions detected for generation 61, two of them contain hundreds of statistically significant SNPs (FDR = 0.01) shared between the generation 22 and 61 data sets. Despite this, those two regions are not among the more differentiated regions in the Manhattan plots (Fig 3A and 3B).

What biological explanations might account for such discrepancies? One possibility is a physiological constraint that eliminates the need for all loci favorable to wheel running to be maintained at high frequencies once a selection limit is reached (see verbal model in [47]). We consider this from the perspective that many complex traits are influenced by hundreds or thousands of loci [63, 64]. Voluntary exercise behaviors would likely be among them, given that they incorporate numerous physiological and morphological traits related to ability (e.g., cardiac muscle, skeletal muscle, bone, metabolism, water and temperature homeostasis) as well as aspects of motivation and reward (e.g., dopamine signaling, chemosensory systems) [65, 66].

Although biological constraints can be defined in various ways [67], in the present context, a constraint would be anything that limits the maximum revolutions that an individual mouse can run during the testing period. Previously, we discussed how different unique responses to identical selection criteria (i.e., “multiple solutions”) could occur and referenced constraints as a potential explanation [47]. To utilize and expand on their example, suppose that mice are subject to a constraint on wheel running caused by joint pain: they stop running when the pain becomes intolerable. In this scenario, joint pain is sufficient to limit wheel running and it serves as a “weak link” or single limiting factor in the biological systems required for high wheel running. Then suppose 10 alleles located at 10 independent biallelic loci, with entirely additive effects, are capable of increasing wheel running. Suppose further that only five such alleles are needed to achieve the maximum amount of wheel running permitted by joint pain. Under this model, if selection acts on a population to increase running, then (1) fixation of the favorable allele at any five of the 10 loci will coincide with a selection limit determined by pain tolerance, (2) none of the alleles at any of the 10 loci must be fixed to reach the pain-determined limit, (3) more than 5 favorable alleles could be maintained at intermediate allele frequencies, and (4) as long as enough favorable alleles are maintained for the selection limit, some favorable alleles can be lost without detriment to wheel running. These factors allow for substantial variation among the replicate lines and considerable flexibility for change within a given line, even for favorable wheel-running alleles at the selection limit. This possibility of "genetic churn" beyond a selection limit that is caused by a physiological constraint also implies that genotype-to-phenotype maps [68–71] may be moving targets and hence difficult to identify. Therefore, we used simulations to compare power to detect and consistency in detected selection signatures, both with and without a physiological constraint.

### Allele frequency divergence over time

The relatively large number of SNPs identified to be divergent between generations 22 and 61 illustrate a shift between the generations. Furthermore, regions with the greatest divergence between the generations align closely with some of the previously identified regions, particularly those identified by the generation 61 mixed model analyses (Table 3). By generation 22, the lines had not had as much time to evolve as much separation in allele frequencies between HR and C as by generation 61. As a result, the differences between the linetypes are generally between -0.5 and 0.5 at generation 22 (Fig 3C), whereas this difference expands to between -1 and 1 for generation 61 (Fig 3D). Given that the significant regions identified at gen 61 are typically going to be those whose difference is near -1 or 1, most likely those same regions at gen 22 had differences within the -0.5 and 0.5 range. Therefore, a growing difference in HR and C allele frequency had to have occurred, in our data, between gen 22 and gen 61 to observe a significant difference at 61. Such loci would naturally be among the more significant in the difference of t-tests over time.

### Simulations comparing power under constrained versus unconstrained models

These simulations were conducted to test whether a physiological constraint on the phenotype of wheel-running behavior could reduce the consistency of loci identified at different generations of a selection experiment. To better simulate realistic phenotypic variance within the population, both the wheel-running and constraint phenotype simulations were based on equations with both genetic and environmental sources of variance, such that both could evolve.

#### Similarities between the constrained model and real data

The constrained model appears to better reflect what we observe in the actual response to selection. This is due to the lack of a clear selection limit achieved under the unconstrained model (Fig 5A). Although the response to selection diminishes over time (likely due to the reduction in heritability: Fig 5E), a clear plateau is not apparent. In contrast—as must be the case—a clear plateau occurs under the constrained model.

#### Correlation between generations 22 and 61 p-values

For the tests comparing allele frequencies at each of 2,096 loci between the HR and C lines, the correlation of p-values between generations 22 and 61 was statistically lower in the constrained model (r = 0.3843) as compared to the unconstrained model (r = 0.3889), though still 4x higher than for the real genomic data (r = 0.0909) (Fig 2). Even with the inclusion of sampling error, the correlation (r = 0.2695) is nearly 3x greater than for the real genomic data, which indicates that other factors (e.g., gene interactions) must be contributing to the differences between the generations.

In spite of the similarity in the correlation of p-values between generations for simulations, the between-generation consistency of detected selection signatures was slightly but statistically greater under the unconstrained (33.3%) than under the constrained model (31.5%). This difference may be due to the constrained model having very slightly (~0.016) though consistently lower selection differentials (Fig 5G versus 5H), which could lead to less fixation of favored alleles. However, the relatively small difference between models in consistency of selection signatures is not enough to explain the large differences in the real data between generations 22 and 61 (Table 2). The inclusion of sampling error into the estimates decreased the 31.5% consistency between generations 22 and 61 differentiated loci to 22.7%. This level of consistency with simulated data remains more than 2-fold higher than for the real data (9.12%), thus implicating the presence of additional factors that reduce consistency in the real data (e.g., epistatic effects).

Overall, our simulations fail to demonstrate why we observe a 17X drop in significant regions from generation 22 to 61 (Table 1), implying instead that we should detect more at generation 61 than at 22 (Table 4).

#### Effect sizes of loci

Under both models, more loci were detected at later generations (Table 4). However, the power to detect loci with the largest effect size was much higher at earlier than later generations (S2 Table and S1 Fig). This pattern makes sense in consideration of the factors that affect the average difference in allele frequency between the HR and control lines and the variance among replicate lines within linetypes. Drift will generally increase the variance among lines with each generation. The allele frequencies in the simulated control lines will be affected only by this drift. Allele frequencies in the HR lines will be affected both by drift and selection, where selection will have stronger effects at loci with larger effect sizes. This results in something of a race between selection increasing the difference in allele frequencies between HR and control lines, while drift increases among-line variance for both HR and control lines. For loci with small effect sizes, drift will have a relatively greater influence over allele frequencies than selection at any generation, and thus detection rates never vary far from the Type I error rate, i.e., power is virtually zero (S2 Table). Loci with large effect sizes, however, are able to differentiate rapidly, often leading to fixation of the favored allele in our simulations (S1 Fig). Even after fixation in the HR lines, drift is still able to increase allele-frequency variance among the control lines (potentially to the point of fixing loci for opposite alleles), thus further reducing the power to detect any differentiation. Thus, the power to detect signatures of selection should increase the most rapidly across generations for loci with the largest effect sizes, but power is also expected to decline after fixation of the favored alleles in the HR lines and with continuing increase in variance among the control lines (S1 Fig).

That the power to detect a locus as differentiated is correlated with its effect size is unsurprising. For example, under the unconstrained model the power to detect selection signatures for loci with 0.4 effect size is about 16.6-fold less than the power to detect loci with 204.8 effect size and 12.8-fold less for the constrained model (generation 22). This gap diminishes to about 7.7-fold difference (both models) by generation 61 (S2 Table), presumably due to the reasons described in the previous paragraph. However, the 0.4 effect size loci are far more numerous than the 204.8 effect size loci (N = 720 and 8, respectively). Consequently, the number of 0.4 effect size loci detected as significant is nearly 5-fold greater (unconstrained) and more than 7-fold greater (constrained) than the number of 204.8 effect size loci detected. The most notable difference between the constrained and the unconstrained models is that at generation 22 the unconstrained model yielded substantially more power than the constrained model for loci with the largest effect sizes (0.724 to 0.485, respectively; unpaired t-test, P = 4.01E-22). This would imply that constraints may have a substantial impact on the ability to detect selection at loci with the greatest effect sizes, a result that deserves further study.

For identifying possible biological functions, we would ideally focus on loci with relatively large effect size, as these will have the most direct influence on the phenotype and may serve as potential targets for future functional studies. We have no information on effect sizes of SNPs or regions detected as differentiated for our real data. The relative proportions of low- and high-effect size loci among the detected selection signatures in the real data will likely vary from our simulations, depending on the actual distribution of those effect sizes and other factors. However, the simulations do suggest that we may have numerous small-effect size loci among our detected selection signatures. The inclusion of the “strict” culling method was meant to prioritize regions that would have large effect sizes. Having more loci that are differentiated and linked together would be expected from those regions under strong selection because recombination would have fewer generations to break up linked base pairs before the region becoming fixed in the HR lines. As the simulations have so many more loci with small effect sizes, at generation 0, when we compare the lowest p-value produced for each simulation for the 0.4 effect size we tend to see lower p-values than loci with 204.8 effect size simply because of more opportunities to produce a low p-value. However, generation 22 appears to be better for detecting a greater proportion of selection signatures from loci with large effect sizes as the relative proportion on large effect size loci appears to be higher (S2 Table).

### Possible biological functions of generation 22 differentiated regions

Ontology analyses identified biological processes that can be grouped into three categories: sensory perception of smell, antifungal innate immune response, and embryonic skeletal system morphogenesis. Of these, the system that is most consistent between generations 22 and 61 is the perception of smell, which was among the mostly clearly differentiated systems at generation 61 [13]. As was discussed by Hillis et al. [13], the experimental procedure for measuring wheel running, for logistical reasons, involved mice being placed on wheel over three batches and mice in batches 2 and 3 are placed on wheels which still smell of the previous mouse [27]. Evidence of an evolutionary response to this is visible in the HR lines in that HR mice will run at very different speeds if on a wheel that is clean, previously traversed by a male, or previously traversed by a female [72]. Alterations in the transcriptome also indicates changes in olfactory and vomeronasal systems [48]. Taken together, these results indicate that perception of smell may be a notable factor in their motivation for running on the wheels and also consistent with the idea that motivation is expected to evolve before ability [20, 73]. Interestingly, although both generations demonstrate evolution in genomic regions association with olfaction and vomeronasal, the regions implicated in each generation are different, with exception of the region on chromosome 14 (chr14:52,115,206–53,776,455), which was identified by the generation 22 WRT analyses and the generation 61 mixed model analyses (Table 2). However, additional studies should be done to address the effects of olfactory/vomeronasal systems more directly on running behavior of the HR mice. This could be done with ablation procedures on HR and C mice and observing changes in running behavior. The antifungal ontology term is possibly a hitchhiker with the vomeronasal genes also present in the differentiated region (chr6:122,815,876–124,446,843).

Ontology analyses also indicated embryonic skeletal system development as result of a group of *Hoxb* genes within a differentiated region. If these *Hoxb* are the driving force underlying the many skeletal differences that have been documented between HR and C lines [38–46], then this is an exciting discovery because it would represent a response to selection in a group of genes known to be evolutionarily influential in body patterning and development [74]. However, whether the *Hoxb* genes are the cause of skeletal differentiation is unclear. Although *Hoxb* genes may play a role in these changes, they are far from the only candidates. GO term “skeletal system development” includes 7 additional non-*Hox* genes, including *Phospho1*, *Col1a1*, and *Mbtd1*, which are all located in the same differentiated region as the *Hox* genes. Furthermore, individual loci demonstrating the greatest differentiation do not appear to be in *Hox* genes themselves or their regulatory regions. Even if *Hox* genes are a hitchhiker in a region with other genes more directly targeted by selection due to their skeletal effects, exploring potential side effects of this evolution would be of interest. Expression analyses during developmental stages when these genes are most active may provide insight into how *Hox* genes may be altered in the HR mice.

#### Other genes of potential interest

The 79 genes included in top regions also contain a few of particular note: *Cited2*, *Rbm24*, and *Dspp*. Each of these genes is associated with ontologies and phenotypes that have been identified as differentiated between the HR and C mice. *Cited2* is a gene whose knockout (KO) has been associated with alterations in brain and heart morphology [75–77] and has also been associated with adrenal development [78]. As noted in the introduction, HR mice have larger brains and hearts than C mice [25, 28, 32, 33]. Additionally, adrenal corticosterone levels were found to be different between the linetypes [73, 79]. *Rbm24* is a gene associated with skeletal muscle fiber differentiation, particularly during regeneration following injury [80–82]. The HR and C lines have demonstrated differences in muscle fiber types within muscles important for wheel running such as the gastrocnemius [35–37]. However, differential response to muscle injury has not been found between the linetypes [83]. Lastly, *Dspp* was identified among the differentiated genes. This gene has been associated with development of long bones (such as femurs) and cortical and trabecular bone thickness [84, 85]. The HR and C mice have shown various differences in bone morphology (see Introduction).

## Limitations and conclusions

Some of the limitations of the present study include trying to compare results of pooled genome sequencing (generation 22) to individual mouse sequencing (generation 61: [13]). Though the alleles of the individual mice can be combined to imitate pooled genome sequences, the differences in number of mice sampled and sampling error make comparisons problematic (see Methods). This is illustrated by the decrease in p-value correlations (between generations 22 and 61) as compared to both the unconstrained and constrained simulations. Nevertheless, as argued above, neither the increase in number of regions detected as differentiated at generation 22 nor the lack of correspondence between detected regions at generations 22 and 61 can be explained solely by methodological differences.

The constraint simulations have their own limitations in that they do not account for male vs female running differences (females run more than males) [3]. In addition, dominance, epistasis, and gene-environment interactions were not considered. The exclusion of these features may be why we were unable to achieve realistic levels of among-line variation, particularly among the High Runner lines. This model also does not include linkage disequilibrium or realistic rates of recombination. Additionally we do not include reduction in breeding success across generations, which may explain the drop in selection differential observed by Careau et al. [3]. Lastly, we did not explore the potential effects of relaxing selection for four generations, as when the mice were moved from Wisconsin to California (see Introduction). A cluster of generations of no selection in the HR lines could allow for some drift of the favored alleles.

Although, we are unsure as to why we see so many regions at FDR = 0.01 that do not correspond to the generation 61 findings by Hillis et al. [13], our simulations suggest that regions with the strongest effect sizes on wheel running are likely to be among the generation 22 regions. Given the statistical significance and number of SNPs identified in our “strictly” culled differentiated regions, these regions are most likely to have had the greatest impact on wheel running at the start of the selection experiment. Among these regions are genes related to olfactory/vomeronasal function, reward pathways, and a miRNA cluster that has been associated with energy homeostasis in neonatal development. All of these associations make sense based on known phenotypic differences between the HR and control lines (see Introduction).

Future directions might include more complex simulations [14, 50, 86], which may better help to explain the 17X increase in regions detected at generation 22. Including genomic data from more generations (especially from the base population, generations near to but before the selection limit, and current generations [i.e., around 100]) may provide more clarity regarding how the response to selection changes across phases of the selection response [62, 87]. Analyses using all loci and a kinship matrix would enable determination of some interactions between genes. Functional analyses, such as knockouts of some of the genes whose alleles appear to have been favored by selection, may provide direct evidence of influence on wheel-running behavior [88–90]. Furthermore, analyses of other physiological aspects of these KO mice may help to better understand the mechanisms by which the gene influences wheel running.

## Supporting information

S1 Fig Simulation power results by effect size and generation. Effect Size—Color: 204.8—brown, 102.4—red, 51.2—orange, 25.6—yellow, 12.8—dark green, 6.4—light green, 3.2—dark blue, 1.6—light blue, 0.8—dark purple, 0.4—light purple.
(PDF)

S1 File Regularized and windowed regularized F-test (WRT). Description of methodology and rationale.
(PDF)

S2 File Parameters and seeds for constraint simulations. List of the parameters and seeds used for the simulations with and without constraints.
(PDF)

S1 Table Effect size and Type I error rates. Includes error rates and means for different effect sizes and sample sizes.
(PDF)

S2 Table Power to detect differentiation between HR and C lines in relation to effect size of locus and generation under two simulation models. Includes effect sizes, sample sizes, revolutions when homozygous, and power and mean for each generation and model.
(PDF)

S3 Table Differentiated regions identified at generation 22 (FDR = 0.01). Includes chromosomal location, size of region, most statistically significant base pair p-value, and position of this base pair.
(XLSX)

S4 Table Genes included in “strict” culling regions at generation 22. (XLSX)

S5 Table Gene ontology results for generation 22 “strict” culling genes. Includes GO terms, fold enrichment, and raw p-values.
(XLSX)
