# Supplementary material for: Large changes in detected selection signatures after a selection limit in mice bred for voluntary wheel-running behavior
Source: PLoS One. 2024 Aug 1;19(8):e0306397. doi: 10.1371/journal.pone.0306397 (PMC11293672; doi:10.1371/journal.pone.0306397)

S1 Fig. Simulation power results by effect size and generation

Effect Size - Color: 204.8 - brown, 102.4 - red, 51.2 - orange, 25.6 - yellow, 12.8 - dark green, 6.4 - light green, 3.2 - dark blue, 1.6 - light blue, 0.8 - dark purple, 0.4 - light purple

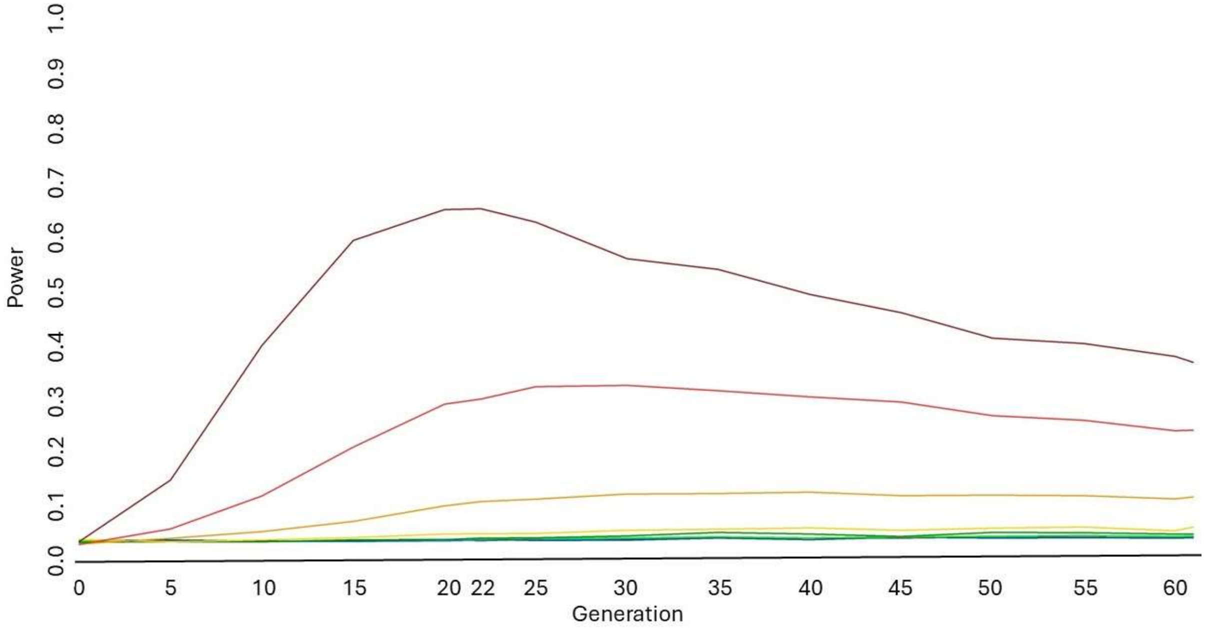

Supplement: S1 Fig — Effect Size—Color: 204.8—brown, 102.4—red, 51.2—orange, 25.6—yellow, 12.8—dark green, 6.4—light green, 3.2—dark blue, 1.6—light blue, 0.8—dark purple, 0.4—light purple. (PDF) [file pone.0306397.s001.pdf]
