# Supplementary material for: Large changes in detected selection signatures after a selection limit in mice bred for voluntary wheel-running behavior
Source: PLoS One. 2024 Aug 1;19(8):e0306397. doi: 10.1371/journal.pone.0306397 (PMC11293672; doi:10.1371/journal.pone.0306397)
Supplement: S1 File — Description of methodology and rationale. (PDF) [file pone.0306397.s002.pdf]

S1 File: Regularized and windowed regularized F-test (WRT)

The regularized T-test has been suggested for genome-wide association studies involving pooled sequencing and low numbers of replicate lines (Baldwin-Brown *et al.* 2014). With 4 replicate lines for each linetype, this technique will not necessarily differ much from an ordinary T-test that uses unequal variance (Baldi and Long 2001). Nevertheless, we performed some comparisons. We compared the t-test (assuming unequal variance) to the standard T-test, the regularized T-test, and the windowed regularized T-test. The standard F-test (T-test squared) is calculated as

$$F = \frac{(x_1 - x_2)^2}{\frac{(v_1 + v_2)}{r}}$$

where x_1 and x_2 are the allele frequency means for each linetype, v_1 and v_2 are their respective variances, and r is the number of replicates per linetype (4 lines). The variance components are calculated with

$$v_i = \frac{1}{4-1}\sum_{j=1}^{4}(\bar{y}_{ij} - \bar{y}_i)^2$$

where i is the linetype, j is the line, and $\bar{y}_i$ is the average allele frequency across all lines within a given linetype. The regularized f-test is similar with the following modification

$$F = \frac{(x_1 - x_2)^2}{(1-w)\frac{(v_1 + v_2)}{r} + 2w\frac{\bar{v}}{r}}$$

where w is the coefficient of regularization and $\bar{v}$ the average variance across all loci and both linetypes (variance calculated for each linetype separately, then averaged). Ultimately, the function of the regularized f-test is to alter the variance component such that the new variance being used is a weighted average of the variance for the locus and the variance for all loci in the genome. w represents the relative weights given to the two variances (larger w gives greater weight for the whole-genome variance).

One of the reasons why the regularization has been shown to perform well with smaller numbers of replicates is that these are subject to relatively large sampling error. Thus, the variance component for several loci may be small due to such sampling error. Regularization of the variance helps to avoid p-values that are inappropriately low (Baldi and Long 2001).

Although we find this rationale reasonable and likely to increase p-values for loci with questionably low variance, it fails to acknowledge that reduced variance is expected for loci under selection as compared to loci subject only to drift. To reclaim some of the power lost with the regularized f-test using $\bar{v}$ as the average across the entire genome, we modified this equation to use $\bar{v}$ as the average across a sliding window of 1 mbp on either side of the locus being analyzed (windowed, regularized T-test or WRT). This is arguably a broad sliding window; however, given that (1) selection can reduce variance over broad areas (Baldwin-Brown *et al.* 2014) and (2) random genetic drift may reduce

variance in some areas of the genome, the broad window should increase p-values for regions affected only by drift more than it would for those affected by selection.
