# Supplementary material for: Large changes in detected selection signatures after a selection limit in mice bred for voluntary wheel-running behavior
Source: PLoS One. 2024 Aug 1;19(8):e0306397. doi: 10.1371/journal.pone.0306397 (PMC11293672; doi:10.1371/journal.pone.0306397)
Supplement: S2 File — List of the parameters and seeds used for the simulations with and without constraints. (PDF) [file pone.0306397.s003.pdf]

S2 File. Parameters and seeds for constraint simulations.

These are the parameters used by the simulations comparing power to detect selection signatures with and without a biological constraint.

N <- 20 # number of mice parenting each generation (half males and half females)

Each of the 10 pairs will produce 1 male and 1 female for controls and "sel" males and "sel" females for HR as shown in next line

sel <- 5 # Number of mice being selected from (for each sex)

c_sel <- 2 # Number of CONTROL mice produced for each sex (should be 1 unless doing diagnostics)

loci <- 2096 # number of loci influencing the trait (total wheel running) NOTE Will create problems with kurtosis if exceeds 4000

start_af <- 0.5 # starting allele frequency for loci (0.25 was picked for maintaining running at about 6000 for controls and allows for a total of 21000)

gen <- 61 # Number of generations

constraint1 <- 50000 # number of revolutions that cannot be exceeded

Ve_constraint <- 1750 # Environmental multiplier for constraint

max_running <- 50000 # Highest running allowed for a mouse, running levels above this are replaced with this value

Min_running <- 100 # Lowest running allowed for a mouse, running levels lower this are replaced with this value

gene_mod <- 1.3 # Multiplier for the genetics variance (larger numbers increases running variance)

Mean_start <- 4570 # Starting population mean (1527.4 according to my calculations based on average gain from dominance [complete, 1/4], 4400 if no dominance)

Ve <- 2100 # Set the multiplier for the environmental variance

dominance <- 0 # amount of dominance 0 to 2 (e.g., 1 for dominant heterozygotes to have 50% genetic effect as homozygous dominant)

dom_proportion <- 1/4 # proportion of loci to be affected by dominance, should be listed as 1/x (e.g., 1/5 for 20% dominance rate)

season <- c(0.769, 1, 1.3, 1) # Vector representing running multiplier in each season c(Summer, Fall, Winter, Spring), not used for gen 0

s_loci <- 100 # Number of loci to influence seasonal response

s_start_af <- 0.5 # Starting allele frequency for seasonal alleles

s_epistatis <- 0 # Set to 1 in order to include selection on seasonal response

```
seasonalVar <- 0 # set equal to 1 if you want seasonal variation by locus

con_loci <- 100 # Number of loci to influence seasonal response

con_start_af <- 0.5 # Starting allele frequency for seasonal alleles

con_epistatis <- 1 # Set to 1 in order to include selection on seasonal response

write_breed <- 1 # set to 1 if you want to write out the breed order or 0 if not
```

For parallelization the code was run in a loop of 10 iterations:

```
for (z in 1:10) {[simulation code]}
```

20 iterations of these loops were ran simultaneously (labeled A through T)

Each iteration had its own seed starting with 42 for A, and increasing by one for each subsequent letter (e.g., 43 for B, 44 for C).
