# Supplementary material for: Large changes in detected selection signatures after a selection limit in mice bred for voluntary wheel-running behavior
Source: PLoS One. 2024 Aug 1;19(8):e0306397. doi: 10.1371/journal.pone.0306397 (PMC11293672; doi:10.1371/journal.pone.0306397)
Supplement: S1 Table — Includes error rates and means for different effect sizes and sample sizes. (PDF) [file pone.0306397.s004.pdf]

S1 Table. Effect size and Type I error rates

| Effect size | N (2096) | Unc. G0 Mean | Unc. G0 Error | C. G0 Mean | C. G0 Error | Average Type I Error |
|---|---|---|---|---|---|---|
| 0.4 | 720 | 30.7 | 0.0426 | 29.2 | 0.0405 | 0.0415 |
| 0.8 | 480 | 19.8 | 0.0412 | 19.5 | 0.0406 | 0.0409 |
| 1.6 | 312 | 13.0 | 0.0416 | 12.6 | 0.0405 | 0.0410 |
| 3.2 | 216 | 8.6 | 0.0396 | 8.8 | 0.0405 | 0.0401 |
| 6.4 | 144 | 5.9 | 0.0410 | 5.7 | 0.0398 | 0.0404 |
| 12.8 | 96 | 4.5 | 0.0467 | 3.9 | 0.0401 | 0.0434 |
| 25.6 | 60 | 2.4 | 0.0407 | 2.5 | 0.0408 | 0.0408 |
| 51.2 | 36 | 1.6 | 0.0433 | 1.4 | 0.0378 | 0.0406 |
| 102.4 | 24 | 1.0 | 0.0396 | 0.9 | 0.0383 | 0.0390 |
| 204.8 | 8 | 0.3 | 0.0338 | 0.4 | 0.0500 | 0.0419 |
