# Supplementary material for: Large changes in detected selection signatures after a selection limit in mice bred for voluntary wheel-running behavior
Source: PLoS One. 2024 Aug 1;19(8):e0306397. doi: 10.1371/journal.pone.0306397 (PMC11293672; doi:10.1371/journal.pone.0306397)
Supplement: S2 Table — Includes effect sizes, sample sizes, revolutions when homozygous, and power and mean for each generation and model. (PDF) [file pone.0306397.s005.pdf]

S2 Table. Power to detect differentiation between HR and C lines in relation to effect size of locus and generation under two simulation models

Effect size	N (2096 Total)	Revolutions when Homozygous	Unc. G22 Mean	Unc. G22 Power	C. G22 Mean	C. G22 Power	Unc. G61 Mean	Unc. G61 Power	C. G61 Mean	C. G61 Power	P-value G22	P-value G61	P-value G22 vs G61 Unc.	P-value G22 vs G61 C.
0.4	720	1.08	27.9	0.039	27.7	0.038	33.4	0.046	34.6	0.048	0.7709	0.1807	1.99E-11	6.90E-14
0.8	480	2.16	19.5	0.041	19.0	0.040	23.9	0.050	22.7	0.047	0.4128	0.0710	1.03E-10	2.32E-08
1.6	312	4.32	12.3	0.039	11.7	0.037	15.4	0.049	14.7	0.047	0.1682	0.2656	4.98E-08	1.37E-08
3.2	216	8.64	8.7	0.040	8.4	0.039	10.5	0.049	9.6	0.044	0.4491	0.0231	3.81E-05	0.0044
6.4	144	17.28	5.4	0.037	5.9	0.041	6.9	0.048	6.7	0.047	0.1010	0.5649	7.40E-06	0.0335
12.8	96	34.56	4.4	0.046	4.1	0.043	6.4	0.066	5.1	0.053	0.4392	0.0001	3.66E-08	0.0006
25.6	60	69.12	3.5	0.059	3.5	0.058	6.8	0.114	3.9	0.065	0.7796	7.08E-18	6.95E-22	0.1076
51.2	36	138.24	4.6	0.128	4.4	0.121	8.4	0.234	4.5	0.124	0.3750	4.22E-25	5.88E-23	0.6808
102.4	24	276.48	10.1	0.421	8.6	0.358	9.0	0.373	6.2	0.257	1.58E-05	2.05E-14	0.0014	8.39E-13
204.8	8	552.96	6.5	0.809	6.3	0.781	3.0	0.373	3.0	0.370	0.1725	0.9175	4.41E-50	1.37E-42

Unconstrained (Unc.) and Constrained (C.) means represent the average number of loci with the given effect size detected at generation 22 (G22) or generation 61 (G61). Power is calculated for each effect size by dividing the mean by the total number loci with that effect size in the simulated genome.

[1]T-test assuming different variances comparing number of loci detected in the unconstrained model vs constrained model at generation 22

[2]T-test assuming different variances comparing number of loci detected in the unconstrained model vs constrained model at generation 61

[3]T-test assuming different variances comparing number of loci detected at generation 22 vs 61 in the unconstrained model

[4]T-test assuming different variances comparing number of loci detected at generation 22 vs 61 in the constrained model
